# Can Mass Trapping Reduce Thrips Damage and Is It Economically Viable? Management of the Western Flower Thrips in Strawberry

**DOI:** 10.1371/journal.pone.0080787

**Published:** 2013-11-25

**Authors:** Clare Sampson, William D. J. Kirk

**Affiliations:** Centre for Applied Entomology and Parasitology, School of Life Sciences, Keele University, Newcastle under Lyme, Staffordshire, United Kingdom; Rutgers University, United States of America

## Abstract

The western flower thrips *Frankliniella occidentalis* (Pergande) (Thysanoptera: Thripidae) is a cosmopolitan, polyphagous insect pest that causes bronzing to fruit of strawberry (*Fragaria* x *ananassa*). The main aim of this study was to test whether mass trapping could reduce damage and to predict whether this approach would be economically viable. In semi-protected strawberry crops, mass trapping of *F. occidentalis* using blue sticky roller traps reduced adult thrips numbers per flower by 61% and fruit bronzing by 55%. The addition of the *F. occidentalis* aggregation pheromone, neryl (*S*)-2-methylbutanoate, to the traps doubled the trap catch, reduced adult thrips numbers per flower by 73% and fruit bronzing by 68%. The factors affecting trapping efficiency through the season are discussed. Damage that would result in downgrading of fruit to a cheaper price occurred when bronzing affected about 10% of the red fruit surface. Cost-benefit analysis using this threshold showed that mass trapping of thrips using blue sticky roller traps can be cost-effective in high-value crops. The addition of blue sticky roller traps to an integrated pest management programme maintained thrips numbers below the damage threshold and increased grower returns by a conservative estimate of £2.2k per hectare. Further work is required to develop the *F. occidentalis* aggregation pheromone for mass trapping and to determine the best timing for trap deployment. Mass trapping of thrips is likely to be cost-effective in other countries and other high-value crops affected by *F. occidentalis* damage, such as cucumber and cut flowers.

## Introduction

Mass trapping of insect pests is used routinely on more than 10 million hectares of commercial crops around the world, predominantly against Lepidoptera, Coleoptera, Diptera and Hemiptera [1]. A variety of lures are used to attract them, including food, colour, kairomones and pheromones, either alone or in combination [2]. Interest in pheromone mass trapping has increased because traps can be species-specific, which reduces the impact on non-target species and because pheromones are active at very low concentrations and do not need to be sprayed directly onto a crop, which is safer for the environment than chemical insecticides. This gives a sustainable pest control strategy that can be integrated with the biologically-based programmes increasingly used. The greatest success has been against pest species that occur at low densities, have limited host ranges, long generation times, isolated populations and low mobility [3]. In contrast, mass trapping is not widely used commercially against thysanopteran (thrips) pests, which are typically polyphagous, have high population densities, short life cycles and rapid population increases [4]. Thrips are difficult to control with trapping, as huge numbers need to be caught in order to make an impact on a population [5]. High densities of sticky traps have reduced *Thrips palmi* (Karny) (Thysanoptera: Thripidae) in pepper and aubergine in Japan [6] and *Frankliniella intonsa* (Trybom) (Thysanoptera: Thripidae) in strawberry [7] and pepper in South Korea [8], but no assessment of crop damage was done in these studies, so there was no evidence of economic viability. Natwick et al. [9] found a negative correlation between trap catch on blue and yellow sticky cards and numbers of *Frankliniella occidentalis* (Pergande) (Thysanoptera: Thripidae) and *Thrips tabaci* Lindeman (Thysanoptera: Thripidae) on lettuce plants, suggesting that mass trapping could cause population reduction. Sticky traps failed to prevent damage from *T. tabaci* in onion [10].

We have used the western flower thrips, *F. occidentalis*, in a commercial strawberry crop to test whether mass trapping can reduce thrips damage. We have also assessed its economic viability because the high density of traps required may be too expensive. It is a polyphagous species that causes economic damage to a variety of crops around the world [11]. In protected and semi-protected (grown in open-sided polytunnels) strawberry, adult and larval *F. occidentalis* cause flower abortion and fruit bronzing resulting in fruit downgrading and crop loss [12], [13]. Strawberry is a high value crop in the UK with a production value of £245m in 2011 [14]. About 3300 ha of strawberry were grown in 2012 [14] of which at least 1000 ha were susceptible everbearer varieties (continuously flowering and fruiting over several months) where 10% losses due to thrips damage were typical (R. Harnden, pers. comm., 2013) as a result of increasing resistance to chemical insecticides [15]. This 10% over 1000 ha equates to about £8m loss due to *F. occidentalis* per annum in UK strawberry and alternative control methods are sought. Sticky traps are used extensively for monitoring thrips, and blue traps are particularly attractive to *F. occidentalis* [9], [16]. Various scents, including *para*-anisaldehyde and methyl isonicotinate, are known to increase thrips trap catch [17], but the identification of the *F. occidentalis* aggregation pheromone, neryl (*S*)-2-methylbutanoate, in male thrips [18], which attracts both female and male adults, offers an opportunity for enhanced mass trapping, which has not previously been tested. The pheromone is currently used for precision monitoring (Thripline _ams_, Syngenta Bioline Ltd, Clacton, UK), but does not have European Union registration as a control method.

The overall aims of this study were to determine whether mass trapping with blue sticky roller traps catches sufficient *F. occidentalis* to reduce thrips damage in semi-protected strawberry, whether there is added benefit of the aggregation pheromone and whether mass trapping is economically viable.

## Materials and Methods

Mr Simon Clarke and Mr George Busby and Sons allowed access to their fields for field studies.

### Efficiency of trapping through a season (2011)

To determine the relative efficiency of trapping through a season, thrips numbers on the crop were compared to weekly pheromone trap catches from 17 May to 18 October 2011 in a 2.4 ha, first-year, semi-protected, commercial everbearer strawberry crop (*Fragaria* x *ananassa* cultivar Camarillo) near Tamworth, UK (52°62’N 1°76’W). The crop was grown in coir grow bags (10 cm×100 cm), each containing 10 plants, on a Mypex mulch (Don and Low Ltd, Forfar, UK) with drip irrigation (Dripnet PC, Netafim Ltd, Tel Aviv, Israel). The bags were spaced to give a density of 10 plants per m^2^. The grower continued with his usual thrips control programme, which included releases of the predatory mite *Neoseiulus cucumeris* (Oudemans) (Acarina: Phytoseiidae) (approximately 100 per plant spread over the season) and two insecticide treatments with spinosad (Tracer, Landseer Ltd, Chelmsford, UK) on 15 and 30 July 2011.

Two blue sticky card pheromone traps (25 cm×10 cm, Russell IPM Ltd, Deeside, UK) were set up in two separate tunnels from first flowering on 17 May (earlier flowers had been de-blossomed as is common commercial practice). Traps were placed 20 m apart and 20 m from the ends of the tunnels to reduce sunlight and edge effects. Traps were placed vertically (south facing, landscape orientation) on metal posts (60 cm) with the bottom edge of the traps about 10 cm above the crop and secured using rubber bands (size 33, Censtretch, Rochester, UK). Pheromone lures (Thripline _ams_, Syngenta Bioline Ltd, Clacton, UK), each containing 30 µg of the *F. occidentalis* aggregation pheromone, neryl (*S*)-2-methylbutanoate, were placed in pheromone lure holders (55 mm long, 25 mm diam., Russell IPM Ltd) slotted over the metal posts using a small wire loop, with the cage hanging on the north side of the trap, shaded from direct sunlight. Traps and pheromone lures were replaced weekly to ensure that the traps did not become contaminated with dirt and other insect species and so that the pheromone release rate was similar between weeks. Collected traps were placed in separate polythene wrappers and stored in a freezer. The numbers of thripids (family Thripidae) on traps were counted under a binocular microscope in the laboratory. Occasional aeolothripid and phlaeothripid thrips were present, but were excluded from the counts as some can be predatory and none are considered to be pests on strawberry. In the rest of this paper the term ‘thrips’ is used to refer to adult thripids (i.e. adult thrips in the family Thripidae). Where larvae are discussed, this is indicated clearly.

Thrips numbers on the crop were assessed weekly by eye on 10 plants within 10 m of each trap. Eye counts were analysed because the results could then be related to grower monitoring which is done in the same way. Plants were selected at random from different grow bags. On each plant, the total numbers of flowers and fruit per plant and the numbers of adult thrips in one medium-aged flower (petals open, and with anthers starting to dehisce [19]) and on one white fruit were counted by eye using a x7 head lens (optiVISOR, LightCraft, London, UK). These eye counts were used to estimate the numbers of thrips per m^2^. The picked flowers were then pooled by placing them in 80% alcohol and all the thrips were examined for species identification (see below). These pooled samples were used to calculate the percentage of *F. occidentalis* in the eye counts. A simple estimate of the numbers of thrips per m^2^ for the purpose of comparison was made by multiplying the mean numbers of adult thrips per flower by the numbers of open flowers on the 10 plants, then adding the mean number of adult thrips per white fruit multiplied by the numbers of fruit (all stages) on the 10 plants. Numbers of adult *F. occidentalis* typically increase as the fruit matures from green to white to red, with about twice as many adult thrips on red fruit compared to those on green fruit [12], so we used an intermediate stage. Although this is an underestimate, as it does not include thrips on leaves or off the plant, it is a relative measure of the thrips population for looking at variation over time. In whole plant counts in different fields during the season, on the cultivar Camarillo, about 1% of *F. occidentalis* adults were found on strawberry leaves compared to flowers and fruit (unpublished data, 2012). A “trapping efficiency index” was calculated each week by dividing the numbers of thrips per trap by the estimated numbers of thrips per m^2^ in order to give a relative measure of the proportion of the population caught by trapping. Thus it can be used to indicate when trapping efficiency was high or low. A data logger (EL-USB-1, Lascar Electronics, Salisbury, UK) was placed in a white delta trap (273 mm length, 130 mm height, œcos, Kimpton, UK) to shade it from the sun and was placed in the crop canopy to record temperature and humidity.

### Mass trapping experiment (2012)

To establish whether mass trapping can reduce thrips damage, a field experiment was carried out in a 12.5 ha second-year, commercial everbearer strawberry crop (cultivar Camarillo) near Stafford, UK (52°75’N 2°22’W). The crop was grown under 40, open-sided, connected polytunnels in raised beds covered in black plastic mulch and irrigated by T-tape (Netafim). Each polytunnel contained five strawberry beds with 10 plants per m^2^ and was 8.5 m wide and 233 m long. Blue sticky roller traps were used in addition to the grower’s usual thrips control programme, which included releases of the predatory mite *N. cucumeris* (fortnightly releases from mid-May to mid-August at 25 mites per plant per release) and three insecticide treatments with spinosad (Tracer) on 18 July, 5 August and 28 August 2012. Blue traps were used as they catch more thrips than yellow traps and a narrower range of non-target species [9], [20]. A data logger was placed in a white delta trap as described above to record temperature and humidity.

On 9 July 2012, the experiment was laid out in a randomised design with three treatments and three replicate plots. Two weeks before the start of the experiment the thrips distribution through the field was surveyed by counting the numbers of adult thrips per medium-aged flower by eye, using a x7 head lens (optiVISOR), in 10 flowers from 10 different 4 m^2^ plots (n = 100) located in a zig-zag pattern across the field. The experiment was then sited in an area of the field that had been shown to have a uniform distribution of thrips. Each plot was 17.6 m long and 8.5 m (one tunnel) wide, with 33 m between plots within tunnels and 25 m between plots in different tunnels. All plots were located at least 40 m in from the ends of the polytunnels to reduce edge effects. Treatments included control plots without any traps or lures, trap plots with blue sticky roller traps (Optiroll, 100 m×30 cm, cut to plot length, Russell IPM Ltd), and pheromone plots with blue sticky roller traps (Optiroll) plus lures containing the *F. occidentalis* aggregation pheromone (Thripline _ams_). The blue sticky roller traps were double-sided and placed down both sides of the treatment plots, clipped onto the polytunnel legs between strawberry beds so that they did not interfere with work on the crop. The base of each trap was level with the crop canopy (approximately 30 cm above the ground). The pheromone lures, each containing 30 µg neryl (*S*)-2-methylbutanoate, were pushed into a hole made in the blue sticky roller trap with a hole punch beside every tunnel leg (2.2 m apart, 18 lures per plot). A spacing of 2.2 m was adopted partly for convenience as the interval corresponded with the spacing of the polytunnel legs and because previous experiments had shown a difference between pheromone and control traps at a spacing as low as 3.4 m [18]. An assessment of adult thrips numbers was made on 9 July 2012 before the traps were put up, then at approximately monthly intervals on 8 August and 10 September. On each assessment date, 40 medium-aged flowers and 20 fully swollen white fruit were sampled regularly from across each plot, excluding 2.2 m in from the ends to reduce edge effects. The assessment of white fruit enabled comparison of the same fruit stage between plots and dates, as red fruit of comparable ripeness was not always available following picking and the selective picking of undamaged red fruit would have biased red fruit samples. The numbers of adult thrips per flower and the numbers of seeds surrounded by bronzing per fruit were counted by eye using a x7 head lens (optiVISOR) as eye counts were considered both effective and reliable and could be related in future to grower counts for monitoring [21]. The picked flowers were then pooled by placing them in 80% alcohol and all the adult thrips were examined for identification (see below). These pooled samples were used to calculate the percentage of *F. occidentalis* in the eye counts. The numbers of flowers per plant were counted on 10 plants from the middle of the trial area on each assessment date.

On 10 September 2012, the numbers of larval thrips per flower were counted by eye as above, at the same time as the adult counts. Such counts should be interpreted with caution as only the larger larvae will be visible and the counts are not as reliable as counts of adults [21]. However, they would give a relative indication of changes in a population.

To test the impact of trapping on thrips density, simple estimates of the numbers of adult thrips per plot (on each sample date) and thrips per roller trap (at the end of the experiment) were made. Numbers of adult thrips per plot were estimated by multiplying the mean numbers of adult thrips per flower by the mean numbers of flowers per plant and plants per plot (set by the planting density). Flower counts, although providing an underestimate of the thrips population, would account for about 74% of the adult *F. occidentalis* population in strawberry (unpublished data from whole plant counts, 2012). The numbers of thrips on roller traps were estimated by counting the total number of thrips on six randomly selected sub-samples (10 cm×30 cm) of blue sticky roller traps per plot, then extrapolating the total numbers of thrips per trap. Both estimates are considered a rough approximation and are not directly comparable as the thrips per plot assessments were ‘snapshots’ while the trap counts were cumulative, and both include thrips that may have flown in from adjacent tunnels. However, the estimates are sufficient to test whether enough thrips are caught on traps to reduce the thrips population.

### Estimation of the damage threshold for downgrading fruit

The damage threshold for the 2012 mass trapping experiment was quantified by comparing the amount of bronzing on 25 higher priced fruit (class 1 fruit) and 25 fruit that had been downgraded to a lower price (class 2 fruit), using harvested red fruit. This was done on 13 September 2012 on cultivar Finesse and on 2 October 2012 on cultivar Camarillo (the same variety as the experiment). While different cultivars may be more or less susceptible to thrips damage, they were graded on the same criteria. There were insufficient thrips to cause downgrading of fruit due to fruit bronzing during July and August in the experimental plots. Fruit was graded and selected by pack-house staff at the site and a few individual fruit were then removed that had been downgraded for reasons other than bronzing, such as size, shape, bruising and disease. Bronzing was quantified on remaining fruit by counting the number of seeds surrounded by bronzing per fruit in the laboratory using a x7 head lens (optiVISOR). To give an indication of where the damage threshold between class 1 and class 2 fruit lay, the inter-quartile ranges of bronzing for both classes of fruit were compared and the threshold was selected from between the lower quartile of class 2 fruit and upper quartile of class 1 fruit, such that the majority of class 2 fruit was above and the majority of class 1 fruit was below.

As fruit bronzing can be caused by environmental factors as well as thrips [22], a regression analysis was used to confirm the relationship between fruit bronzing and thrips density using mean bronzing on white fruit and mean thrips per flower in the nine plots in the mass trapping experiment in September 2012.

The damage threshold from the pack-house was assessed on red fruit whereas the plot data were obtained from white fruit (where damage shows up more easily), so a conversion factor was required to predict the damage threshold on white fruit. To quantify the conversion factor, bronzing was assessed on white fruit in 32 sub-plots of 0.5 m^2^ on 5 September 2012, then on red fruit (before picking) in the same plots on 10 September (when the white fruit from 5 September had turned red). On each date, the numbers of seeds surrounded by bronzing were assessed by eye, using a x7 head lens (optiVISOR), on five fruit per plot. The conversion factor was quantified by regressing bronzing on white fruit (from 5 September) on bronzing on red fruit (from 10 September) using a square root transformation to normalise the data.

### Calculation of the economic returns of mass trapping

The economic returns to growers from trapping were calculated by subtracting the total cost of trapping per hectare (including blue sticky roller traps, pheromone lures and the cost of labour to erect the traps once for the period July to September) from the estimated increase in fruit sales per hectare during September (estimated sales in treated plots minus estimated sales in control plots). The weight of fruit sold (class 1 and class 2 combined, in kg per ha) was assumed to be the same for all treatments and was based on the actual yield per ha in the crop for the month of September, although this may have underestimated the weight in treated plots as a slightly higher weight may be associated with lower thrips numbers [13]. This would have underestimated the return on trapping. Earlier months were discounted as there were insufficient thrips to cause fruit downgrading. The damage threshold, derived from harvested red fruit with the white fruit conversion (see above), was used to calculate the proportion of class 1 and class 2 fruit in each plot. Fruit sales were then projected based on the proportion of class 1 and class 2 fruits and sale price of each. Prices for class 1 and class 2 fruit were taken from an average of grower prices from five supermarket buyers for cultivar Camarillo in 2012.

### Thrips identification

The flower samples collected during the field monitoring in 2011 and the mass trapping experiment in 2012 were rinsed in alcohol to remove the adult thrips. *Frankliniella occidentalis* was separated from other species by eye under a binocular microscope, then sub-samples of thrips were placed on microscope slides in Hoyer’s solution to confirm the identification under a compound microscope. Confirmation of the identification (on slides) was carried out on 100 randomly selected *Frankliniella* spp. and 100 randomly selected *Thrips* spp. per month.

### Statistical analysis

Data for analysis of variance were transformed to log_10_(*x*+1) to homogenise the variance. Data for regressions were transformed to log_10_ *x* or square root, as appropriate to normalise the variance. Residuals were checked for normality. Multiple comparisons used Tukey’s test. Tables and figures show untransformed means for adult thrips per flower, seeds per fruit and thrips per trap. The percentage reduction in thrips numbers and fruit damage between each treatment and the control in the mass trapping experiment was calculated by comparing the untransformed means. Statistical analysis was carried out with Minitab 16 (Minitab Inc., USA).

## Results

### Efficiency of trapping through a season (2011)

**Figure 1:**
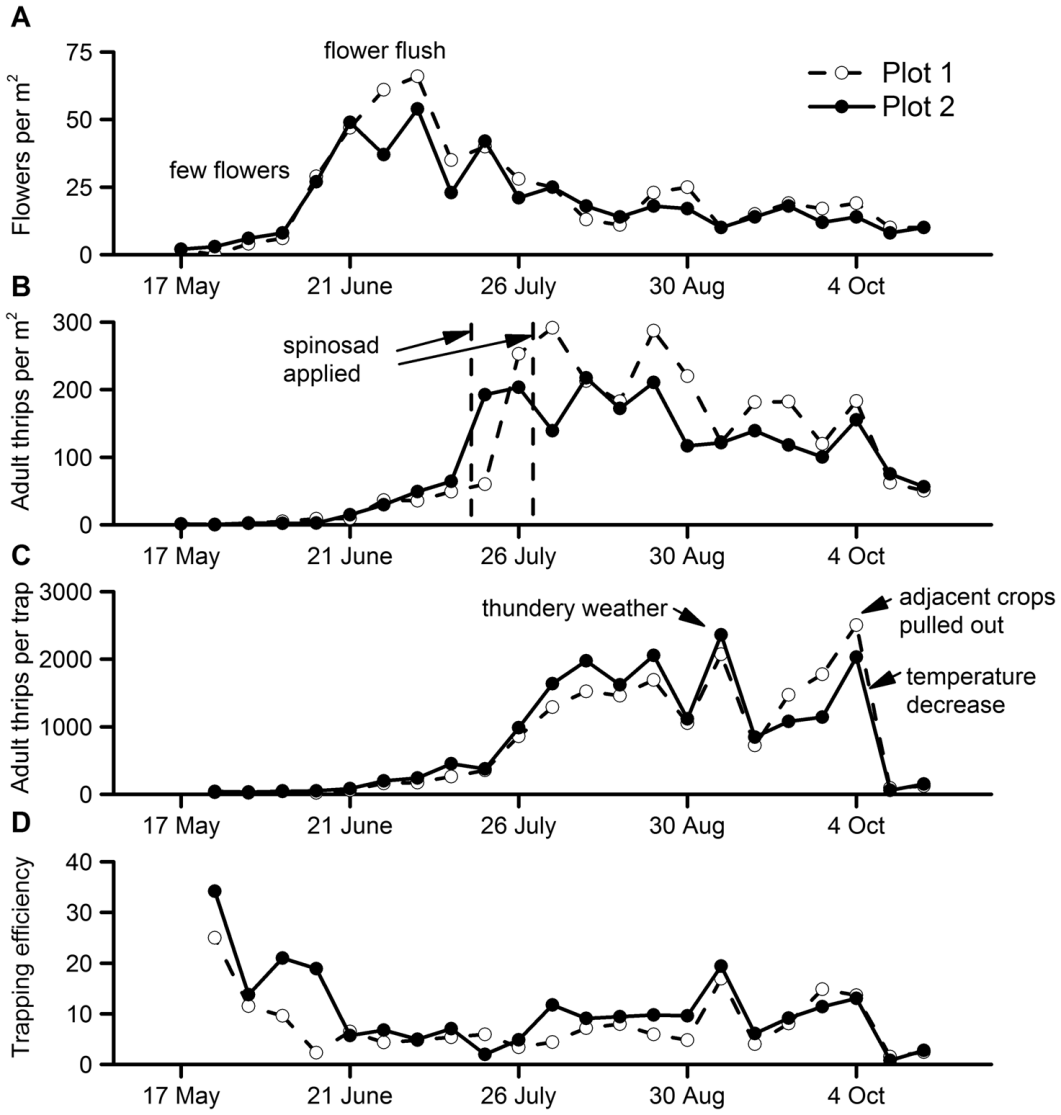
Seasonal changes in a semi-protected strawberry crop in 2011. (A) flowers per m^2^, (B) adult thrips per m^2^, (C) adult thrips per pheromone trap, and (D) trapping efficiency index (thrips per pheromone trap divided by thrips per m^2^) in two plots.

Thrips numbers increased steadily from when the strawberry plants came into flower in mid-May (Figures 1A, B), then rapidly in the second half of July and remained high for the rest of the season until October, when they declined again. Blue sticky card pheromone traps caught large numbers of thrips, averaging over 800 thrips per card trap per week (mid-May to mid-September) but exceeding 2000 thrips per card trap per week on occasions. Each card trap caught the number of thrips per week equivalent to all the adult thrips in an area of about 9 m^2^ of crop, on average. Trapping efficiency was greatest in the brief period between mid-May and early-June, when flower numbers were below 10 per m^2^ and adult thrips numbers were low (Figure 1D). The trapping efficiency dipped from mid-June to late July when the crop was in full flower flush (30-70 flowers per m^2^, Figure 1A) and thrips numbers still low (Figure 1B), but increased to intermediate levels throughout the main cropping period (August-September), when flowers averaged about 20 per m^2^ and adult thrips numbers were >4 per flower (Figure 1D). A spike in trap catch at the end of August occurred during thundery weather (Figure 1C) [23], and a second spike in trapping occurred at the end of September when a strawberry crop in the adjacent field was being pulled out during warm weather (max 33°C) (Figure 1C). The sharp drop in trapping efficiency at the end of the season corresponded with falling temperatures (from a weekly mean maximum of 31.6°C to 20.1°C) in early October.

### Mass trapping experiment (2012)

**Figure 2:**
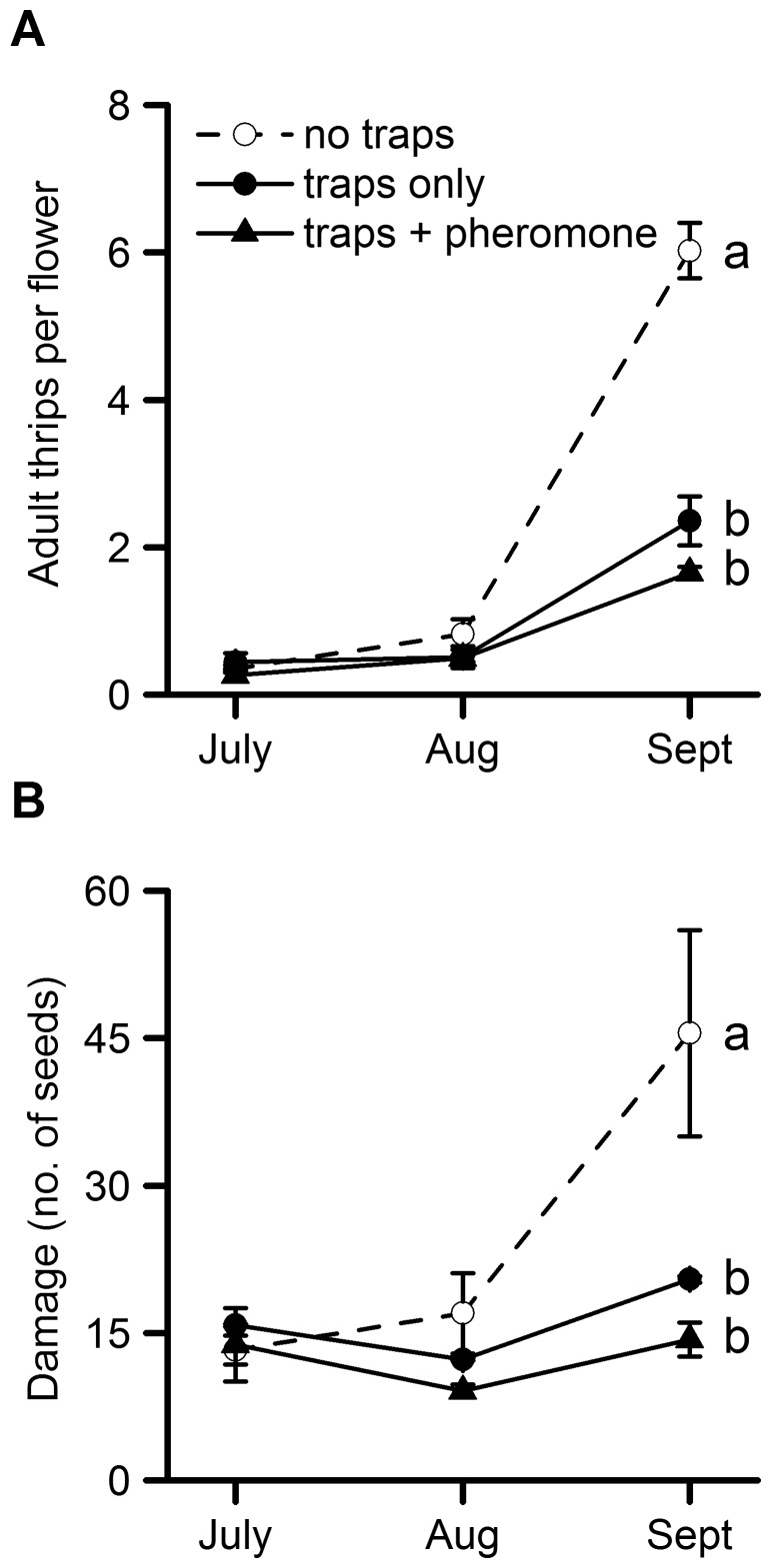
The effect of mass trapping on thrips numbers and fruit damage. Samples taken on 9 July, 8 August and 10 September 2012 in: plots without blue sticky roller traps; plots with blue sticky roller traps only; and plots with blue sticky roller traps and pheromone lures. (A) mean adult thrips per flower ±SE and (B) mean fruit damage ±SE. Damage was recorded as the number of seeds surrounded by bronzing on swollen white fruit. Differences were significant in September (thrips numbers *P*<0.001, fruit damage *P* = 0.006) (*n* = 3). Means with the same letter are not significantly different (*P*>0.05).

Thrips were well controlled until mid-August. As adult thrips numbers in the plots without traps were low in early-August (<1 per flower), it was not possible to show a reduction in adult thrips numbers (*F*_(2,6)_ = 1.55, *P* = 0.29) or fruit damage (*F*_(2,6)_ = 3.29, *P* = 0.11) with trapping at this time (Figure 2). The thrips population took-off in August when the population was largely *F. occidentalis* (see thrips identification below), which was not well controlled by spinosad (Tracer). Mass trapping with blue sticky roller traps alone, or with additional *F. occidentalis* aggregation pheromone, reduced adult thrips numbers by 61% and 73% (*F*_(2,6)_ = 60.1, *P*<0.001) and fruit damage by 55% and 68% respectively (*F*_(2,6)_ = 13.29, *P* = 0.006) by early September (Figure 2). Maximum day-time temperatures exceeded 20°C on all but two days during the experiment, which was sufficient for *F. occidentalis* flight, which is needed for trapping [24].

Counts of thrips larvae by eye on 10 September showed that mass trapping with blue sticky roller traps alone, or with additional *F. occidentalis* aggregation pheromone, reduced larval thrips numbers by 63% and 90% respectively (*F*_(2,6)_ = 14.1, *P* = 0.005). The mean number of larvae per flower (±SEM) was 1.83±0.49 on control plots without traps, 0.68±0.15 on plots with traps alone and 0.19±0.07 on plots with traps and pheromones. Tukey’s test showed that plots with traps alone and plots with traps with pheromone had significantly fewer larvae than the control plots (*P = *0.044 and *P = *0.005 respectively), but that the difference between the two treatments was not significant (*P = *0.17).

To get a relative measure of the impact of trapping on the adult thrips population, trap catch (cumulative total) in September and thrips density (on each assessment date) were compared. The traps were estimated to have caught 13,376 ± 476 (without pheromone) and 25,754 ± 1,844 (with pheromone) thrips per plot. Thus, the addition of the *F. occidentalis* aggregation pheromone to blue sticky roller traps approximately doubled the trap catch (*F*_(1,4)_ = 64.2, *P*<0.001). The numbers of adult thrips on plants were estimated as 924±137 (July), 2,228±566 (August) and 13,255±832 (September) per plot in plots without traps; 1,166±308 (July), 1,387±402 (August) and 5,188±734 (September) per plot in trap plots; and 704±230 (July), 1,341±120 (August) and 3,630±188 (September) per plot in pheromone trap plots. Pheromone traps had accumulated nearly 26,000 thrips per plot over three months, at a time when numbers of thrips per plot were about 13,000 in the plots without traps. The numbers confirm that trapping would have a considerable impact on the population.

### Estimation of the damage threshold for downgrading fruit

**Figure 3:**
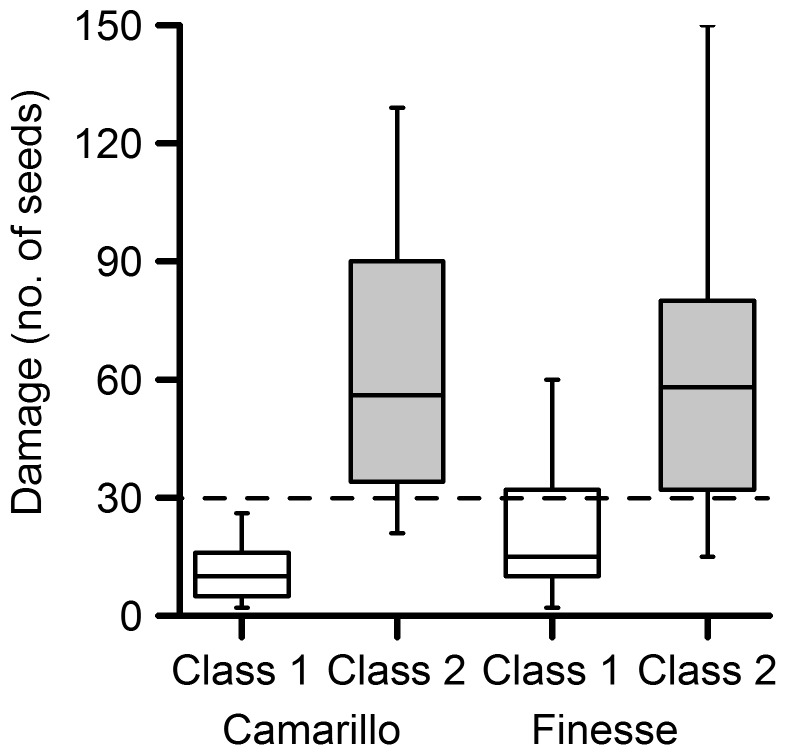
The damage threshold between class 1 and class 2 fruit. Boxplots of the amount of damage on class 1 (empty bars) and class 2 (shaded bars) strawberry red fruit for cultivars Camarillo and Finesse from the pack-house in 2012, suggesting a damage threshold around 30 seeds (dashed line). Damage was recorded as the number of seeds surrounded by bronzing at harvest. Bars indicate the inter-quartile range (50% of values) and the horizontal line indicates the median. The vertical bars indicate the data range excluding any outliers.

Harvested red fruit that had been downgraded to class 2 in the pack-house showed significantly more bronzing than class 1 fruit (*F*_(1,45)_ = 51.4, *P*<0.001, cultivar Camarillo; *F*_(1,48)_ = 32.5, *P*<0.001, cultivar Finesse) (Figure 3). A regression of mean bronzing on white fruit on the mean numbers of thrips per flower in the different plots in September 2012 was consistent with bronzing being caused by thrips (*F*_(1,7)_ = 36.4, *P*<0.001, *r* ^2^ = 82%) (*y*  =  0.98 + 0.84 *x*; where *y*  =  log white fruit bronzing and *x*  =  log adult thrips per flower). The amount of bronzing on class 1 and class 2 fruit pointed to a threshold of bronzing around 30 seeds per red fruit which was about 10% of the fruit surface bronzed (Figure 3). Commercial grading of fruit is done quickly by eye, so there is not a precise threshold between class 1 and class 2 and 10% surface bronzing was considered a reasonable estimate of the damage threshold as >75% of class 1 red fruit had bronzing below this threshold and >75% of class 2 red fruit had bronzing above this threshold (both cultivars combined).

A regression of white fruit bronzing on red fruit bronzing was significant (*F*_(1, 30)_ = 56.3, P<0.001; *r* ^2^  =  64%) (*y*  =  1.10 + 0.97 *x*; where *y*  =  square root of white fruit bronzing and *x*  =  square root of red fruit bronzing). Using this equation, a threshold of bronzing of 30 seeds on red fruit was equivalent to bronzing of around 41 seeds on white fruit, which occurred when there were about six thrips per flower according to the regression equation above. The amount of bronzing was rather similar between fruit stages in this experiment and so the accuracy of conversion of white fruit damage scores to red fruit damage scores made little difference to the cost-benefit analysis.

### Calculation of the economic returns on trapping

The cost of treating every tunnel with blue sticky roller traps was based on 13 traps x 100 m at £25 per trap, plus labour costs based on three workers taking 1.5 days per hectare (£252 per ha at £7 per hour wages) and the cost of pheromone monitoring lures at £2.34 per lure (Table 1). Different (possibly lower) prices are likely to apply if the pheromone was formulated for mass trapping and registered as a control method. Fruit sales were calculated based on £2.99 per kg for class 1 fruit and £1.21 per kg for class 2 fruit and a total yield of 6,262 kg per ha during September (for all treatments). The percentage of class 1 and class 2 fruit, the projected fruit sales in treated and untreated plots using the damage threshold of bronzing of 41 seeds per white fruit, and the return on trapping investment are shown in Table 1. The cost-benefit analysis demonstrates a return on investment for mass trapping for both blue sticky roller traps and blue sticky roller pheromone traps. If the return on investment of £2,200 per ha (Table 1) is typical, then mass trapping could save UK strawberry growers over £2m per annum, projected over the 1000 ha of everbearer strawberry varieties that are susceptible to *F. occidentalis* damage (R. Harnden, pers. comm., 2013).

**Table 1:** Cost-benefit analysis of mass trapping in semi-protected strawberry.

	No traps	Traps only	Traps with pheromone
Total cost of trapping (£ ha^−1^)	-	577	1,953^a^
Sales of class 1 fruit (£ ha^−1^) (%)	12,482 (67%)	17,162 (92%)	18,098 (97%)
Sales of class 2 fruit (£ ha^−1^) (%)	2,525 (33%)	631 (8%)	252 (3%)
Total sales in September (£ ha^−1^)	15,007	17,793	18,351
Return on trapping (£ ha^−1^)^b^	-	2,209	1,391^a^

Comparison of use of no traps with use of blue sticky roller traps, with and without the *Frankliniella occidentalis* aggregation pheromone in a UK crop.

a Cost and return were calculated using current prices of pheromone monitoring lures. If the pheromone were registered and formulated as a control for mass trapping, different prices would apply.

b Total sales with treatment minus total sales without treatment minus cost of treatment.

### Thrips identification

**Figure 4:**
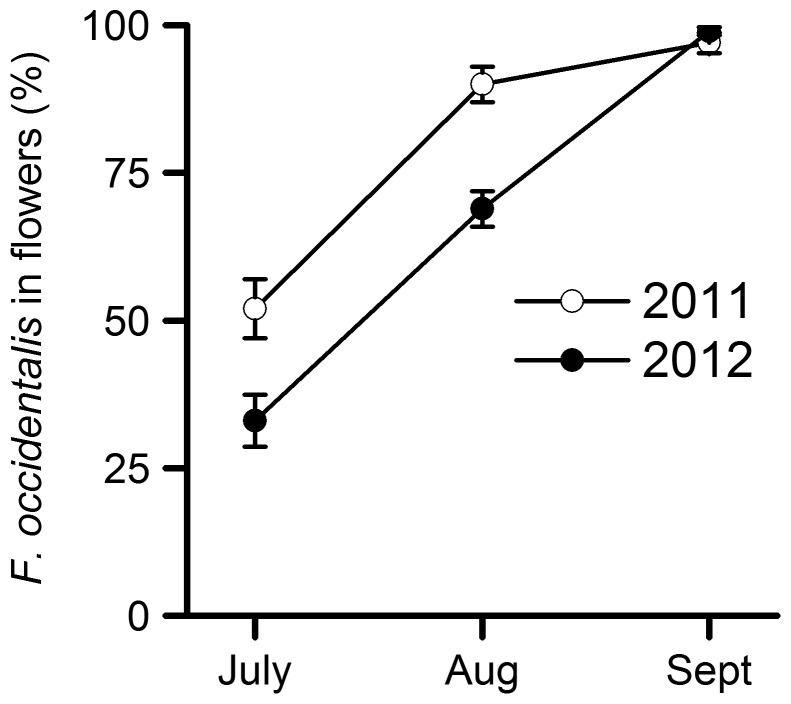
Thrips species composition through the season in strawberry. The percentage of adult thripids in semi-protected strawberry flowers that were *Frankliniella occidentalis* in 2011 and 2012 (*n*>100 for all samples). The error bars are the standard error of the proportion from the sample.

The proportion of *F. occidentalis* in strawberry flowers increased through the season to over 95% by the end of August in 2011 and 2012 (Figure 4). Other thripid species present included *Thrips major* Uzel, *T. tabaci*, *Thrips fuscipennis* (Haliday), *F. intonsa, Frankliniella tenuicornis* Uzel, *Thrips angusticeps* Uzel and *Thrips atratus* (Haliday).

## Discussion

This study confirms that mass trapping can reduce thrips numbers, as found previously in strawberry [7] and pepper [8]. However it goes further than previous work by demonstrating that the reduction in thrips numbers reduces crop damage and that it can also increase grower economic returns. Further work is needed to optimise the timing of trapping and to make the best use of the aggregation pheromone.

Field monitoring gave an indication that trapping could be effective throughout the season, as blue sticky card pheromone traps caught many thrips in every week that the polytunnel covers were in position (mid-May to late-October) (Figure 1C). The efficiency of trapping was similar throughout the season, but possibly better at the start before the first flower flush (Figure 1D). *Frankliniella occidentalis* flight is likely to increase when flowers are scarce because they feed on pollen [25] and would be searching for food. In addition, starved thrips are known to fly more and have a greater response to colour and odour than satiated thrips [26]. This could explain the increased trapping efficiency before the crop had come into full flower. The number of thrips per flower could also play a part by increasing dispersal at high density [27]. There was no suggestion from the data that pheromone trap efficiency declined when adult populations were highest in August. A decline might be predicted if there was a strong effect of competing calling insects on the crop. The broad increases in trap catch and trapping efficiency when maximum temperatures were around 30°C and reduction when maximum temperatures were around 20°C (October) is in line with published information on *F. occidentalis* flight, which showed no take-off at 15°C and increasing flight activity between 20–30°C in a UK population [24]. Trapping efficiency is likely to decline with the length of time that traps are up, as traps become contaminated by dirt and insects and because the glue loses its stickiness in places. In our mass trapping experiment, thrips could be seen walking over some areas of the blue sticky roller traps after two months in the field. Mass trapping was used to protect the crop when it was at most risk of thrips damage (July to September), but the trapping efficiency index suggests that there might be a benefit in mass trapping from the start of the season (from May), as soon as the polytunnels are erected. This is predicted by some mass trapping models [28], although it was not apparent in our data. However, the returns would not be as high early in the season if thrips numbers remain below the damage threshold through May and June. Further work is needed to test the impact of using traps over a more extended period and to determine whether the traps should be replaced during the season.

The addition of the *F. occidentalis* aggregation pheromone to blue sticky roller traps approximately doubled the trap catch in semi-protected strawberry. This is the first published record of using the aggregation pheromone in strawberry and the increase in trap catch is consistent with that found in protected pepper [18], [29], cucumber [30], tomato [31], and top fruit [32], where addition of the pheromone resulted in trap catch increases between 20% and 300%. Increases in *F. occidentalis* trap catch have also been found using plant volatiles and their analogues [33]–[36], which attract other thrips species as well as *F. occidentalis*, so they may be useful for mass trapping in crops where there is a complex of thrips pests. The aggregation pheromone has advantages over plant volatiles where *F. occidentalis* is the main thrips pest species as very small quantities are required to elicit a response [37] (<0.5 g per ha was used in this study) and it increases *F*. *occidentalis* trap catch without directly affecting key natural enemies [29], [32]. Although a mix of thrips species was present at the beginning of the season in strawberry (Figure 4), fruit damage occurred when the thrips population was predominantly *F. occidentalis* (August and September), which is when the aggregation pheromone is likely to be most effective.

As well as reducing the adult thrips population, trapping reduced the larval thrips population by a broadly similar amount, taking into consideration the greater variability of larval counts. Although both adults and larvae can cause damage to strawberries, larvae are considered the most damaging [12] and our results suggest that the reduction in adult population led to fewer larvae, which then led to less damage.

The damage threshold defined in our study equated to a density of around six adult thrips per flower in the field, which was within the range of published damage thresholds in strawberry [12], [38] when predatory mites are present [39]. The economic returns calculated using the damage threshold (Table 1) are considered conservative as they do not include loss of fruit that occurred in late August and October, nor do they consider possible consequential benefits such as reduced spraying costs or reduced numbers of thrips in the following season. In this proof of principle study we used a high density of pheromone monitoring lures (every 2.2 m), but if the lures could be spaced at wider intervals without losing efficacy, then the economic returns on trapping would have been greater than with traps alone. Further work is required on the range of pheromone attraction in thrips to optimise the spacing and formulation of traps and lures for mass trapping.

We have shown that mass trapping can be cost-effective against a polyphagous, high-density pest species with a short generation time, and integrates well with a pest management programme for *F. occidentalis* in semi-protected strawberry. The cost-benefit calculations, while done in the UK, are likely to be applicable to other countries and other high-value crops such as cucumber and cut flowers.
